# Serum soluble immune checkpoint levels predict cervical lymph node metastasis of differentiated thyroid carcinoma patients

**DOI:** 10.1002/cam4.6382

**Published:** 2023-07-27

**Authors:** Yi Shao, Xinru Gui, Yuxin Wang, Lei Sheng, Dong Sun, Qingdong Zeng, Huayang Wang

**Affiliations:** ^1^ Department of Thyroid Surgery, General Surgery Qilu Hospital of Shandong University Jinan China; ^2^ Department of Clinical Laboratory Qilu Hospital of Shandong University Jinan China; ^3^ Department of Gastrointestinal Surgery Shandong Cancer Hospital and Institute, Shandong First Medical University and Shandong Academy of Medical Science Jinan China

**Keywords:** differentiated thyroid carcinoma, immune checkpoints, lymph node, metastasis, TIM‐3

## Abstract

**Background:**

Cervical lymph node metastasis (CLNM) is common in patients with differentiated thyroid carcinoma (DTC); however, the efficiency to distinguish CLNM before surgery is limited. T cell exhaustion, characterized by the overexpression of immune checkpoints, plays a critical role in the immune evasion of tumors. The aim of this study is to analyze the association between serum levels of soluble immune checkpoints (sICs) and CLNM in DTC patients.

**Methods:**

Levels of sICs in serum of 71 DTC patients and 56 healthy volunteers were analyzed by ELISA. Peripheral blood mononuclear cells and cervical lymph nodes of DTC patients were isolated and their expression of sICs were analyzed. Lymphocytes in cervical lymph nodes were analyzed for immune checkpoints expression and transcription of exhaustion‐associated factors. 30 out of 71 DTC patients were followed up from 3 to 9 months after the operation, and postoperative sTIM‐3 were analyzed.

**Results:**

Four sICs, including LAG‐3, PD‐1, PD‐L1, and TIM‐3, were increased in DTC patients. All four sICs exhibited higher sensitivity at discriminating CLNM than cervical ultrasound. In the patient‐matched comparison, higher sTIM‐3 levels were observed in tumor‐involved lymph nodes (TILNs) than in normal lymph nodes (nLNs). T lymphocytes in TILNs had higher TIM‐3 surface expression and increased secretion of sTIM‐3 than those in patient‐matched nLNs. Finally, postoperative serum sTIM‐3 levels were decreased in DTC patients with CLNM compared to their preoperative levels.

**Conclusion:**

Serum levels of sICs, especially sTIM‐3, could help to predict CLNM and provide evidence for surgical decision‐making in DTC.

## Graphical abstract

In this study, we analyzed serum levels of soluble immune checkpoints (sICs) and their association with CLNM in patients with DTC. We found that serum sICs analyzed in our study showed higher sensitivity and specificity in CLNM prediction than cervical ultrasound, and high sTIM‐3 levels was an independent risk factor for predicting CLNM. The results implied that serum levels of sICs, especially sTIM‐3, could help to predict CLNM and provide evidence for surgical decision‐making in DTC.

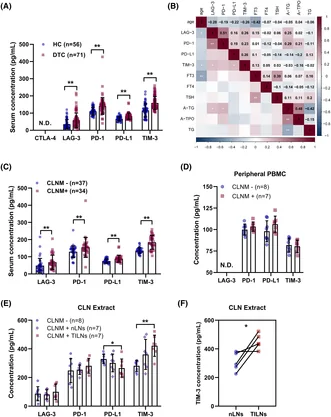

## 1 INTRODUCTION

Thyroid cancer is the most common endocrine malignancy, and its incidence has been increasing steadily over the past decades.^1^ Differentiated thyroid carcinoma (DTC), including papillary thyroid carcinoma (PTC) and follicular thyroid carcinoma (FTC), account for more than 90% of all thyroid cancers. Although DTC has a relatively good prognosis, local invasion might occur in the early stages of the disease, most commonly in locoregional lymph nodes (LNs). In clinical studies, the probability of LN metastasis in patients with DTC ranged from 30% to 60%.^2^, ^3^, ^4^ For patients with LN metastasis at diagnosis, over 50% eventually developed local or distant recurrence.^5^ Preoperative ultrasound is the most common method for diagnosing LN metastasis; however, it has poor sensitivity for the diagnosis of central cervical LN metastasis (CLNM).^6^ Due to the high incidence of central CLNM and its low diagnostic efficacy, prophylactic central cervical LN dissection is recommended by many countries including China.^7^, ^8^, ^9^ However, it increases risk of parathyroid gland and recurrent laryngeal nerve injury. Therefore, a novel preoperative diagnostic method for LN metastasis is important for surgical management and prognosis prediction of patients with DTC.

Owing to its indolent nature, regional LN metastasis of DTC can persist for years without progression or distant metastasis. As a result, tumor cells can coexist with antitumor immunity for a long time without being eliminated by immune cells, suggesting that the antitumor immune response may be compromised. T‐cell exhaustion is a special dysfunctional state due to long‐term antigen stimulation, characterized by the sustained upregulation of immune checkpoint receptor expression, gradual loss of cytotoxicity, and ultimately clonal deletion.^10^ Exhausted T cells were first described in chronic viral infection; however, recent studies have found varying degrees of T‐cell exhaustion in multiple types of cancers due to the continuous stimulation of tumor antigens.^11^ T‐cell exhaustion is an important immune evasion mechanism in cancer. Zhu et al. reported an exhausted state in circulating T cells of the peripheral blood of PTC patients, which is an efficient biomarker to distinguish PTC from thyroid nodules.^12^ Severson et al. found that T cells in the metastatic LNs of patients with DTC exhibited different degrees of exhausted phenotype, demonstrated as upregulated expression of programmed cell death (PD‐1), T‐cell immunoglobulin, and mucin domain‐containing protein 3 (TIM‐3), and CD69; reduced secretion of TNF‐α and IFN‐γ; and weakened cytotoxicity of tumor cells.^13^ The above findings suggest that T‐cell exhaustion may be an important mechanism in LN metastasis in DTC.

Immune checkpoints, such as PD‐1, TIM‐3, cytotoxic T lymphocyte‐associated antigen‐4 (CTLA‐4), and lymphocyte activation gene‐3 (LAG‐3), are upregulated on the surface of exhausted T cells, inhibiting the effect of T cells and driving their apoptosis. Soluble immune checkpoints (sICs) can be released via shedding of the cell membrane or alternative cleavage. The upregulation of sIC is observed in various types of tumors.^14^ Although their pathophysiological significance is poorly understood and remains controversial, the overexpression of sIC before treatment generally indicates the severity of the disease and tolerance against antitumor immunity.^15^ Therefore, sIC may be an effective biomarker for predicting tumor progression and prognosis. However, limited studies have been conducted with DTC patients.

In the present study, we analyzed the serum levels of sIC in DTC patients. The overexpression of sIC before treatment, especially sTIM‐3, was found to indicate a higher probability of CLNM. Further, DTC patients with CLNM had a higher proportion of TIM‐3‐positive T lymphocytes in their tumor‐involved LNs, but not in peripheral blood, and T lymphocytes in DTC‐involved LNs secreted higher levels of sTIM‐3. Finally, serum sTIM‐3 levels were found to decrease in DTC patients with CLNM after the operation. Altogether, serum sIC levels could help to predict CLNM and provide reference for surgical decision‐making in DTC.

## 2 MATERIALS AND METHODS

### 2.1 Patients and histopathologic parameters

This study was conducted in accordance with the Declaration of Helsinki and approved by the Human Research Ethics Committee of Qilu Hospital of Shandong University (China). All patients gave informed consent for sample collection and the subsequent analyses. Patients who were recruited had thyroid nodules detected during neck ultrasound examination and confirmed as malignant through fine‐needle aspiration (FNA) biopsy. These patients underwent thyroidectomy at Qilu Hospital of Shandong University in 2022. The choice of surgical approach follows guidelines of Chinese Society of Endocrinology and China Anti‐Cancer Association.^7^ In brief, the choice of surgical approach (total thyroidectomy, near‐total thyroidectomy or lobo‐isthmectomy) depends on factors including tumor diameter, location, bilateral multifocality, presence of evident extrathyroidal extension, clinical lymph node metastasis, and family history. All patients underwent central neck dissection. Therapeutic lateral neck dissection was performed for patients with preoperative FNA confirmation, radiographic suspicion of nodal involvement (cN1b), and/or intermediate/high recurrence risk. DTC, central and/or lateral CLNM was confirmed via postoperative histopathology. Patients with recurrent thyroid cancer or other histopathological types, except DTC, were excluded. The clinical data of patients were shown as Supplemental Table S1. Patients with a history of other malignancies were also excluded. Healthy controls were individuals who underwent physical examinations at our hospital and denied any chronic disease history. No obvious abnormalities were identified during physical examination.

### 2.2 Sample acquisition and processing

Preoperative serum samples were collected following laboratory testing. LNs were collected and processed according to a previously published protocol, with some modifications.^13^ Briefly, enlarged LNs ranging from 0.9 to 1.7 cm in diameter were bisected. Thereafter, the interior tissue section was subjected to histopathology to confirm the presence of CLNM and the posterior section was processed to generate a single‐cell suspension or LN extract. To generate a single‐cell suspension, LNs were washed with cold Hanks balanced salt solution (HBSS), digested with 2.5 mg/mL Liberase™ TL and 400 U/mL collagenase D (Roche) on an orbital shaker at 37°C and 80 rpm for 10 min, and disrupted at room temperature via gentle pipetting up and down 50 times, followed by an additional 10 times. The reaction was terminated by placing the digest on ice and adding 10 mL cold RPMI‐1640 with 10% FBS. Single‐cell suspensions were extracted from connective tissue, spun down at 450 g for 5 min at 4°C, and resuspended in erythrocyte lysis buffer (Solarbio). The cells were cryopreserved in freezing solution (Invigentech). Under some conditions, CD3^+^ T lymphocytes were isolated from the single‐cell suspension of LNs using anti‐CD3‐conjugated magnetic microbeads (Miltenyi Biotech) according to the manufacturer's instructions.

The LN extract was generated for ELISA according to the protocol recommended by Abcam. Briefly, LN tissues with similar weights (approximately 5 mg) were homogenized using an electric homogenizer (Scientz‐48 L) in 800 μL complete extraction buffer (100 mM Tris, 150 mM NaCl, 1 mM EDTA, and 1% Triton X‐100). Thereafter, the homogenized tissues were centrifuged at 13,000 rpm for 20 min at 4°C. The supernatant was collected and stored at −80°C for further analysis.

Peripheral blood mononuclear cells (PBMCs) were isolated from the peripheral blood of DTC patients using gradient media (TBDsciences). The isolated PBMCs were cryopreserved as described above.

### 2.3 Cell culture

PBMCs or CD3^+^ T lymphocytes from LNs were resuspended in complete RPMI‐1640 with 1:100 penicillin/streptomycin and 200 U/mL recombinant human IL‐2 (rhIL‐2, R & D) and seeded in a 24‐well plate at a density of 1 × 10^6^/mL. After 7 days, the supernatant was collected for further analysis.

### 2.4 Enzyme‐linked immunosorbent assay (ELISA)

The levels of human CTLA‐4 (Abcam, Cat: ab264616), LAG‐3 (Abcam, Cat: ab193707), PD‐1 (Abcam, Cat: ab252360), PD‐L1 (Abcam, Cat: ab277712), and TIM‐3 (Abcam, Cat: ab231932) in serum or cell culture supernatants were quantified using commercial ELISA kits according to the manufacturer's instructions.

### 2.5 Flow cytometry

Cells isolated from LNs were stained with anti‐CD3‐PE (Elabscience), anti‐CD4‐PerCP (BD Biosciences), anti‐CD3‐PerCP (Elabscience), anti‐CD8‐PE (BD Biosciences), anti‐PD‐1‐FITC (Biolegend), or anti‐TIM‐3‐FITC (Biolegend) antibodies. The isotype controls were analyzed in parallel. The samples were processed on a FACSCalibur flow cytometer (BD Biosciences) and analyzed using the FlowJo software.

### 2.6 Quantitative RT‐PCR

RNA from LNs was extracted using TRIzol Reagent (Invitrogen). cDNA was synthesized via reverse transcription. Quantitative RT‐PCR (RT‐qPCR) was performed using a QuantStudio 5 instrument (Applied Biosystems). GAPDH was used as an internal control. The primer sequences were as follows: Tbx21, forward: 5′‐CGA GAT TAC TCA GCT GAA AAT TGA T‐3′, reverse: 5′‐TGT CAA CAG ATG TGT ACA TGG ACT‐3′; Tcf7, forward: 5′‐CTG CCA TCA ACC AGA TCC T‐3′, reverse: 5′‐GCT CAT AGT ACT TGG CCT GCT‐3′; Tox, forward: 5′‐TTC TCT GTG TCA CCC CAT GA‐3′, reverse: 5′‐TCT GGC ATC ACA GAA ATG GA‐3′; and GAPDH, forward: 5′‐TCG GAG TCA ACG GAT TTG GTC GTA‐3′, reverse: 5′‐CTT CCT GAG TAC TGG TGT CAG GTA‐3′.

### 2.7 Statistical analysis

All statistical analyses were conducted using SPSS software version 13.0. Statistical significance was set at *p* < 0.05. Student's *t*‐test or Mann–Whitney nonparametric *t*‐test was used for statistical comparisons, depending on whether the data passed the normality test. The correlation between the two variables was determined using Pearson's correlation.

## 3 RESULTS

### 3.1 sIC is upregulated in the serum of PTC patients pre‐operative

**FIGURE 1:**
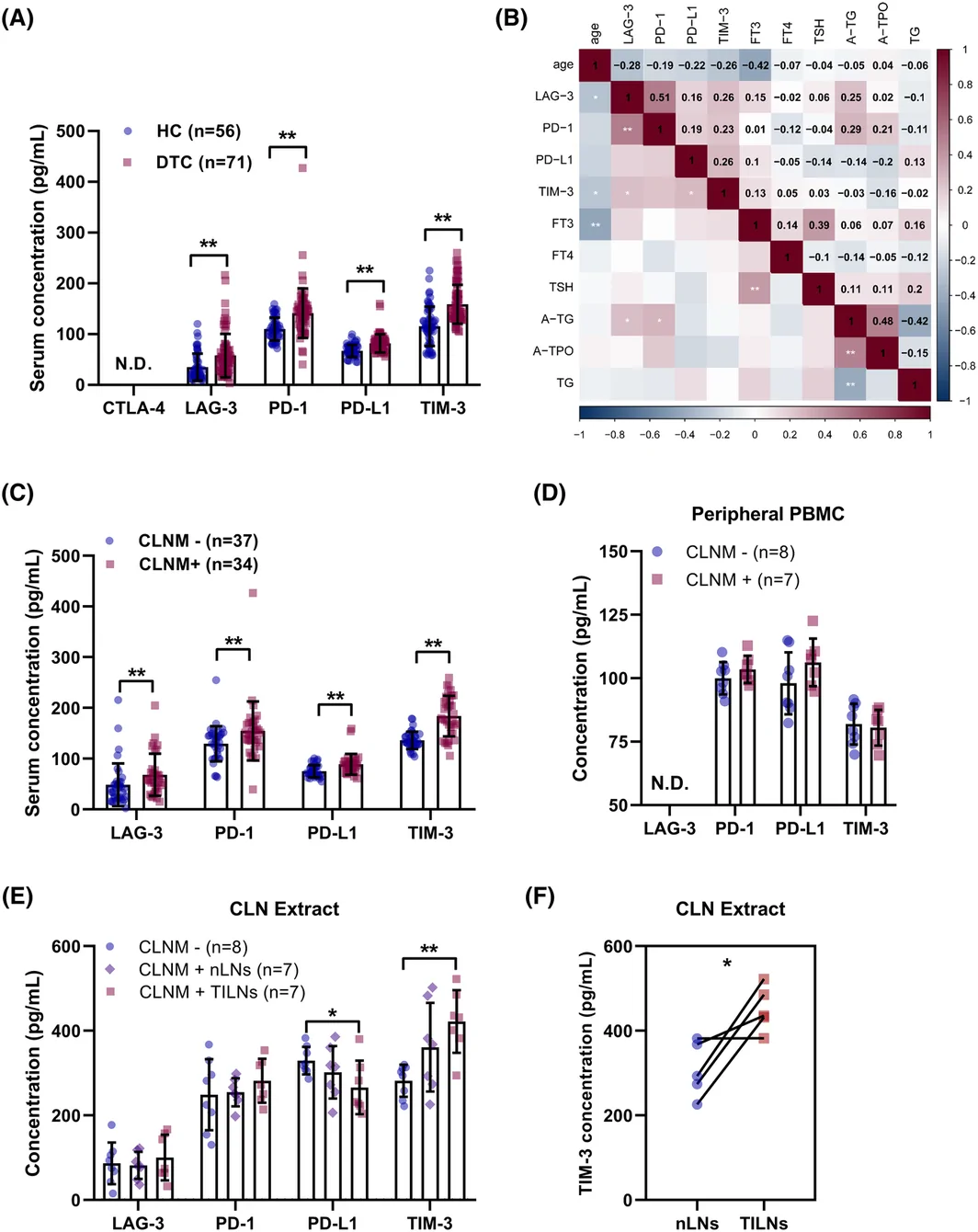
Serum sIC levels were elevated in TILNs of DTC patients. (A) Serum concentration of CTLA‐4, LAG‐3, PD‐1, PD‐L1, and TIM‐3 in healthy controls (HC, *n* = 56) and DTC patients (DTC, *n* = 71). N.D., not determined. (B) Correlation between serum sIC levels and thyroid function results in DTC patients. The number in each cell in the upper right part of the matrix represents correlation coefficience, and the asterisk in the cell in the lower left part of the matrix represents a significant difference in *p* value. (C) Serum concentration of LAG‐3, PD‐1, PD‐L1, and TIM‐3 in DTC patients with CLNM (CLNM+, *n* = 34) or without CLNM (CLNM ‐, *n* = 37). (D) sIC levels in the supernatant of PBMCs of DTC patients with CLNM (CLNM+, *n* = 7) or without CLNM (CLNM ‐, *n* = 8). N.D., not determined. (E) sIC levels in extract from LNs of DTC patients without CLNM (CLNM ‐, *n* = 8), normal LNs of DTC patients with CLNM (CLNM+ nLNs, *n* = 7) or tumor‐involved LNs of DTC patients with CLNM (CLNM+ TILNs, *n* = 7). (F) TIM‐3 levels in extract from patient‐matched nLNs and TILNs (*n* = 5). *p < 0.05; **p < 0.01.

The levels of sICs, including sCTLA‐4, sLAG‐3, sPD‐1, sPD‐L1, and sTIM‐3, in the preoperative serum of patients with PTC were determined by ELISA. Among the five immune checkpoints, four, except for sCTLA‐4, were detected in the serum (Figure 1A). The preoperative levels of sIC in patients with PTC were higher than those in healthy controls, with matched age and sex ratios (Figure 1A). Weak positive correlations were found between the levels of four sICs, especially between sLAG‐3 and sPD‐1, while weak negative correlations were found between the levels of sIC and the age of PTC patients (Figure 1B). There was no correlation between sIC levels and laboratory results for thyroid function in patients with PTC (Figure 1B).

We noticed an overexpression of sIC in DTC patients with CLNM (Figure 1C); therefore, we analyzed the relationships between the clinicopathological parameters of PTC patients and sIC levels. As shown in Table 1, patients with a larger tumor diameter (>10 mm), CLNM, and a higher number of involved cervical lymph nodes (>5) had higher sIC levels. Compared to DTC patients with low risk of recurrence, DTC patients with intermediate risk of recurrence exhibit elevated levels of sTIM‐3 and marginally increased levels of sLAG‐3 and sPD‐1. The levels of sIC had no relationship with tumor subtype, unifocality/multifocality, Hashimoto's thyroiditis background, or BRAF^V600E^ mutation status.

**TABLE 1:** Serum levels of sIC of DTC patients with different clinicopathological parameters.

clinicopathological parameters	Concentration (mean, 95% CI)			
	LAG‐3 (pg/mL)	PD‐1 (pg/mL)	PD‐L1 (pg/mL)	TIM‐3 (pg/mL)
Gender				
Male (*n* = 19)	54.48 (38.50–70.47)	151.9 (115.0–188.7)	86.94 (82.15–91.74)	158.7 (139.0–178.5)
Female (*n* = 52)	59.27 (46.49–72.06)	137.6 (128.3–147.0)	79.92 (74.43–85.40)**	159.1 (148.5–169.6)
Age (year)				
<55 (*n* = 56)	59.39 (48.68–70.09)	141.3 (127.4–155.1)	82.99 (77.97–88.02)	160.8 (150.4–171.3)
≥55 (*n* = 15)	52.79 (23.48–82.1)	142.2 (121.5–162.9)	77.32 (79.91–84.73)	152.0 (131.5–172.5)
Thyroid cancer subtype				
Classical papillary (*n* = 66)	57.23 (46.91–67.56)	142.6 (130.4–154.9)	81.66 (77.18–86.14)	159.9 (150.5–169.3)
Follicular variant of papillary (*n* = 5)	67.99 (−1.066–137.1)	125.8 (86.33–165.2)	83.63 (67.96–99.30)	147 (92.84–201.2)
Tumor diameter (mm)				
Microcarcinoma (≤10) (*n* = 52)	54.81 (42.58–67.05)	137 (122.5–151.5)	78.96 (74.42–83.50)	153.3 (143.3–163.2)
>10 (*n* = 18)	71.11 (51.57–90.64)	157.1 (138.4–175.8)*	89.67 (80.09–99.24)**	179.1 (158.7–199.5)*
Censored (*n* = 1)				
Multifocality				
Yes (*n* = 57)	58.34 (46.27–70.41)	142.3 (128.3–156.3)	79.58 (76.27–82.89)	155.5 (145.8–165.2)
No (*n* = 13)	58.56 (40.34–76.78)	138.1 (120.4–155.7)	93.11 (74.69–111.5)	170.6 (143.8–197.5)
Censored (*n* = 1)				
Hashimoto's thyroiditis				
Yes (*n* = 28)	62.67 (45.35–79.98)	146.7 (135.0–158.4)	83.5 (75.98–91.01)	152.5 (137.9–167.2)
No (*n* = 43)	55.93 (43.15–68.71)	138.5 (120.7–156.3)	81.53 (76.36–86.69)	160.9 (148.8–172.9)
Lymph node involvement				
Non (*n* = 37)	48.56 (34.46–62.65)	129.3 (117.7–140.9)	75.28 (71.24–79.33)	136 (130.3–141.7)
Central neck nodes only (*n* = 20)	60.83 (43.43–78.24)	155.0 (120.5–189.6)	89.09 (80.67–97.51)**	188.9 (170.9–206.9)**
Lateral neck nodes (*n* = 14)	78.87 (52.34–105.4)**	154.1 (140.2–168.1)**	88.59 (74.71–102.5)*	176.9 (152.6–201.3)**
Lymph node number				
Non (*n* = 37)	48.56 (34.46–62.65)	129.3 (117.7–140.9)	75.28 (71.24–79.33)	136 (130.3–141.7)
1–5 nodes positive (*n* = 25)	65.26 (50.11–80.41)*	153 (125.1–180.9)	87.1 (79.81–94.39)**	186.6 (171.1–202.1)**
>5 nodes positive (*n* = 9)	76.59 (35.19–118.0)*	159.3 (148.8–169.8)**	93.85 (73.02–114.7)*	176.6 (140.2–213.0)*
TNM stage^30^				
I (*n* = 68)	58.38 (47.91–68.84)	141.3 (129.3–153.3)	82.03 (77.63–86.42)	156.7 (147.7–165.8)
II (*n* = 3)	49.24 (−31.64–130.1)	144.4 (83.02–205.9)	76.57 (64.48–88.66)	209.8 (145.7–273.8)*
Recurrence risk stratification^7^				
Low (*n* = 38)	51.67 (37.97–65.37)	137.5 (117.9–157.1)	77.97 (72.26–83.68)	142.3 (133.9–150.7)
Intermediate (*n* = 29)	68.26 (51.16–85.37)^a^	145.4 (132.4–158.4)	85.84 (79.10–92.57)^b^	179.5 (164.3–194.7)**
High (*n* = 4)	43.58 (10.12–77.04)	150.0 (124.1–176.0)	88.82 (58.74–118.9)	168.9 (71.64–266.2)
BRAF^V600E^ status				
Wild (*n* = 7)	63.85 (33.80–93.89)	120.8 (83.48–158.2)	78.58 (63.90–93.26)	141.8 (119.4–164.2)
Mutation (*n* = 45)	57.34 (43.32–71.36)	136.8 (128.0–145.5)	82.61 (76.63–88.59)	159.2 (147.7–170.7)
Censored (*n* = 19)				

*Note*: *p < 0.05; **p < 0.01; compared with the first category.

^a^ *p* = 0.05.

^b^ *p* = 0.06.

### 3.2 Role of sIC levels in the preoperative prediction of CLNM in PTC patients

As the above analysis revealed that PTC patients with CLNM have higher sIC levels, we further explored whether sIC could be used as a preoperative predictor of CLNM in PTC patients. First, the ROC curve was analyzed to determine the best cutoff value for each sIC. As shown in Table 2 and Figure S1, the area under the curve (AUC) values of sLAG‐3, sPD‐1, sPD‐L1, and sTIM‐3 were 0.70, 0.69, 0.74, and 0.85, respectively. Under the optimal cutoff value, sTIM‐3 performed best at distinguishing CLNM in all four sICs, with a sensitivity of 73.53% and specificity of 91.9%. In contrast, the sensitivity and specificity of ultrasound for diagnosing CLNM were 44.1% and 83.8%, respectively (Table 2).

**TABLE 2:** The performance of sIC and cervical ultrasound to distinguish CLNM of DTC patients.

	Cutoff value (ng/mL)	AUC	Sensitivity (%)	Specificity (%)
LAG‐3	50.26	0.7	67.65	72.97
PD‐1	145.99	0.69	55.88	75.68
PD‐L1	85.63	0.74	58.82	83.78
TIM‐3	156.14	0.85	73.53	91.89
Ultrasound	‐	0.64	44.12	83.78

Abbreviation: AUC, area under curve.

Univariate analysis was then performed with the demographic characteristics and laboratory examination results of patients. As shown in Table 3, PTC patients with higher sLAG‐3, sPD‐1, sPD‐L1, or sTIM‐3 levels had a 4.94‐, 3.42‐, 6.12‐, and 22.92‐fold higher probability of CLNM than those with lower levels, respectively. In addition, patients who were older (≥55 years old) at the time of diagnosis had a lower probability of CLNM (HR = 0.20), while male patients, higher TI‐RADS scores (five and above), and suspicious involvement of cervical lymph nodes suggested by ultrasound had a higher probability of CLNM (HR = 3.20, 3.42, and 4.08, respectively). When lateral CLNM was distinguished separately from central CLNM, only higher TI‐RADS scores (HR = 5.50) and suspicious cervical lymph nodes suggested by ultrasound (HR = 34.0) were predictive factors of the presence of lateral CLNM.

**TABLE 3:** Univariate analysis of clinical features and laboratory results in discrimination CLNM of DTC patients.

Variants	CLNM		Central/lateral CLNM	
	hazard ratio (95%CI)	*p* value	hazard ratio (95%CI)	*p* value
Demographic features				
Age (years)				
<55	1.00		1.00	
≥55	0.20 (0.05–0.79)	0.02	0.69 (0.06–8.47)	0.77
Gender				
Female	1.00		1.00	
Male	3.20 (1.05–9.75)	0.02	2.33 (0.56–9.64)	0.24
Ultrasound features				
TI‐RADS score				
IV	1.00		1.00	
V and above	3.42 (1.27–9.23)	0.02	5.50 (1.16–26.14)	0.03
Suspicious CLNM				
No	1.00		1.00	
Yes	4.08 (1.35–12.32)	0.01	34.0 (4.91–235.6)	<0.01
Laboratary results				
A‐TG				
Normal	1.00		1.00	
High	1.10 (0.29–4.20)	0.89	2.45 (0.35–17.08)	0.36
A‐TPO				
Normal	1.00		1.00	
High	1.34 (0.40–4.48)	0.63	2.27 (0.42–12.27)	0.34
TG				
Normal	1.00		1.00	
High	0.83 (0.28–2.43)	0.74	1.60 (0.32–7.89)	0.56
Soluble immune checkpoints				
sLAG‐3				
Low	1.00		1.00	
High	4.94 (1.81–13.52)	<0.01	4.91 (0.86–27.88)	0.07
sPD‐1				
Low	1.00		1.00	
High	3.42 (1.27–9.23)	0.02	1.80 (0.44–7.31)	0.41
sPD‐L1				
Low	1.00		1.00	
High	6.12 (2.1–17.84)	<0.01	0.54 (0.13–2.17)	0.38
sTIM‐3				
Low	1.00		1.00	
High	22.92 (6.32–83.03)	<0.01	0.45 (0.1–2.12)	0.31

A multivariate analysis was performed with all significant variants in the univariate analysis. Based on the results, only age and sTIM‐3 was an independent factor at predicting CLNM, with an HR of 0.08 and 48.35, respectively (Table S2).

### 3.3 sTIM‐3 is overexpressed in tumor‐involved cervical lymph nodes and not in PBMCs

To identify the sources of sIC, we first isolated PBMCs from the peripheral blood of PTC patients with or without CLNM and cultured them in vitro. As shown in Figure 1D, PBMCs had low levels of sIC. Further, no difference was found between PTC patients with CLNM and those without CLNM. By analyzing the levels of sIC in locoregional lymph nodes, sTIM‐3 was found to be significantly overexpressed in tumor‐involved lymph nodes (TILNs) (Figure 1E). When sTIM‐3 levels in extracts from cervical lymph nodes were compared, sTIM‐3 levels were upregulated in TILNs compared to patient‐matched normal lymph nodes (nLNs) (Figure 1F).

### 3.4 Lymphocytes in TILNs are more inclined to display exhaustion profile and secrete higher levels of sTIM‐3 than those in nLNs

**FIGURE 2:**
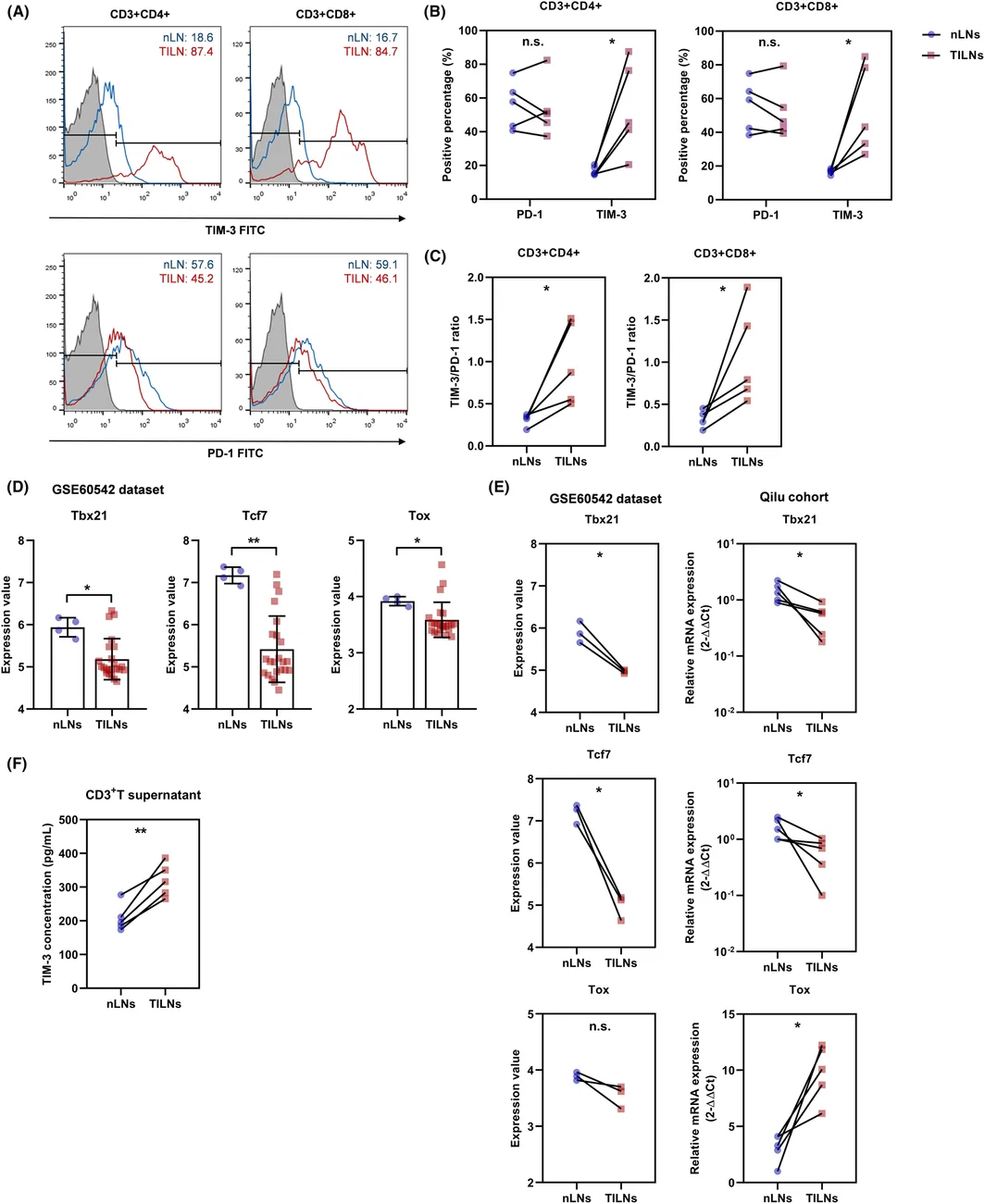
Phenotypes and sTIM‐3 secretion of T lymphocytes in LNs of DTC patients. (A) Typical flow‐cytometry result of TIM‐3 and PD‐1 surface expression on CD3^+^CD4^+^ T subset and CD3^+^CD8^+^ T subset in TILNs (red) and patient‐matched nLNs (blue). (B) Positive percentage of PD‐1 and TIM‐3 on CD3^+^CD4^+^ T subset and CD3^+^CD8^+^ T subset in TILNs and patient‐matched nLNs (*n* = 5). n.s., not significant. (C) Ratio of TIM‐3 to PD‐1 positive percentage in CD3^+^CD4^+^ T subset and CD3^+^CD8^+^ T subset in TILNs and patient‐matched nLNs (*n* = 5). (D) Tbx21, Tcf7, and Tox values in nLNs (*n* = 4) and TILNs (*n* = 23) analyzed by open‐accessed dataset GSE60542. (E) Tbx21, Tcf7, and Tox values in TILNs and patient‐matched nLNs of dataset GSE60542 (left, *n* = 3) and of our cohort (right, *n* = 5). (F) sTIM‐3 concentration in the supernatant of CD3^+^ T lymphocytes isolated from TILNs and patient‐matched nLNs (*n* = 5). *p < 0.05; **p < 0.01.

As sTIM‐3 was overexpressed in the extract of TILNs, we isolated lymphocytes from cervical lymph nodes and analyzed TIM‐3 expression in T‐cell subsets. PD‐1 and TIM‐3 reflect different phases of T‐cell exhaustion (Tex).^11^ The expression of TIM‐3 was significantly upregulated in the CD3^+^CD4^+^ and CD3^+^CD8^+^ T‐cell subsets of TILNs compared with patient‐matched nLNs (Figure 2A,B). In contrast, PD‐1 expression was not significantly different between the T‐cell subsets of TILNs and nLNs (Figure 2A,B). Consequently, the ratio of TIM‐3 to PD‐1, which represents the later exhaustion phase (intermediate or terminal phase), was increased in different T‐cell subsets of TILNs compared to those of patient‐matched nLNs (Figure 2C).

We proceeded to examine the expression of exhaustion‐associated transcription factors. First, we analyzed transcriptomes from the open‐access dataset, GSE60542, which contained four nLNs and 23 TILNs. Based on the results, Tbx21, Tcf7, and Tox were downregulated in TILNs (Figure 2D). However, in the patient‐matched comparison, only Tbx21 and Tcf7 were significantly downregulated (Figure 2E). We verified these results in our cohort. Consistent with the transcriptome dataset, Tbx21 and Tcf7, which indicate progenitor and intermediate Tex subsets, respectively, were downregulated in TILNs compared with patient‐matched nLNs. However, Tox, which indicates the terminal Tex subset, was upregulated in TILNs (Figure 2E).

CD3^+^ T cells were isolated from the TILNs and nLNs and cultured in vitro. After 7 days, sTIM‐3 was found to be significantly overexpressed in the supernatant of CD3^+^ T cells from TILNs compared to those from patient‐matched nLNs (Figure 2F).

### 3.5 Serum sTIM‐3 level is decreased in PTC patients with CLNM postoperative

**FIGURE 3:**
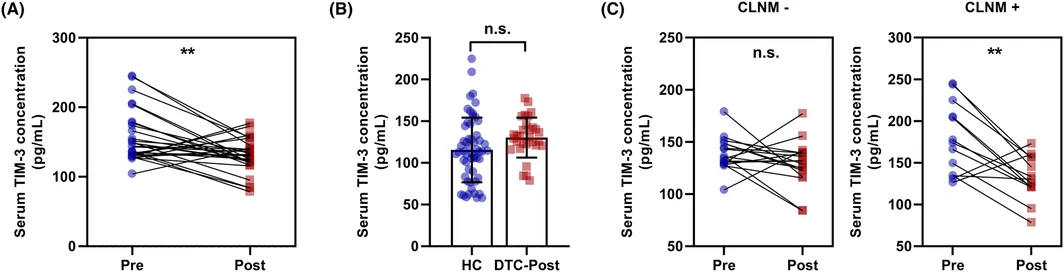
Serum sTIM‐3 levels were decreased after surgery in DTC patients with CLNM. (A) Patient‐matched serum sTIM‐3 levels before surgery (pre) and 3 to 9 months after surgery (post, *n* = 30). (B) Serum sTIM‐3 levels in healthy controls (HC, *n* = 56) and DTC patients after surgery (DTC‐post, *n* = 30). n.s., not significant. (C) Patient‐matched serum sTIM‐3 levels before surgery (pre) and 3 to 9 months after surgery (post) in DTC patients without CLNM (CLNM ‐, left, *n* = 17) and in DTC patients with CLNM (CLNM+, right, *n* = 13). n.s., not significant. **p < 0.01.

As sTIM‐3 secretion was increased in TILNs, we further investigated whether surgical dissection could affect serum sTIM‐3 levels in patients with PTC. Thirty patients in the cohort were followed up from 3 to 9 months after the operation, and postoperative serum was collected. As shown in Figure 3A, sTIM‐3 levels were decreased in postoperative serum compared with patient‐matched preoperative serum. Post‐operative serum sTIM‐3 levels in patients with PTC did not differ from those in healthy controls (Figure 3B). Finally, patients with PTC were further divided according to the presence or absence of CLNM. In patients without CLNM, postoperative serum sTIM‐3 levels were not significantly altered from the pre‐operative level. However, in PTC patients with CLNM, the postoperative sTIM‐3 levels were significantly downregulated (Figure 3C).

## 4 DISCUSSION

Lymph node metastasis is common in DTC, and its preoperative diagnosis is not satisfactory. T‐cell exhaustion is an important immune evasion mechanism in cancer. Previous studies revealed increased exhaustion of T cells in the peripheral blood and cervical lymph nodes of patients with DTC. In the present study, we examined the levels of sIC, whose overexpression is a characteristic of T‐cell exhaustion, in the serum of DTC patients and evaluated their association with the clinicopathological parameters of DTC. Serum sIC levels were found to be elevated in DTC patients, and their overexpression indicated a higher risk of CLNM. Among these, serum sTIM‐3 levels independently predicted CLNM. We also observed overexpression of sTIM‐3 and a higher proportion of TIM‐3^+^ T lymphocytes in tumor‐involved LNs (TILNs) than in normal LNs. Based on the expression of transcription factors, T lymphocytes in TILNs exhibited a switched exhaustion phase from the progenitor or intermediate to terminal phase, and secreted higher levels of sTIM‐3. Finally, serum sTIM‐3 levels were decreased in DTC patients with CLNM at 3–9 months after the operation.

The upregulation of sIC has been found in various types of tumors, including renal cell carcinoma, hepatocellular carcinoma, and non‐small‐cell lung cancer.^16^ However, only few studies have been conducted on DTC. In the serum of DTC patients, four of the five tested sICs, except sCTLA‐4, were upregulated compared to levels in healthy controls. However, due to the large individual variations in sIC in healthy controls, their capacity to assist in diagnosing DTC was limited as only 15.5% of sLAG‐3, 36.6% of sPD‐1, 28.2% of sPD‐L1, and 22.5% of sTIM‐3 in DTC patients were higher than the 95% percentile of those in healthy controls. Nevertheless, the serum levels of the four sICs were significantly higher in DTC patients with larger tumor diameter, CLNM, and a higher number of metastatic lymph nodes, suggesting that they may be associated with higher recurrence risk. sTIM‐3 was the most significantly upregulated sIC in DTC patients with CLNM. AUC analysis revealed that the sensitivity and specificity of sTIM‐3 to distinguish DTC with/without CLNM were 73.5% and 91.9%, respectively, which were markedly higher than those of the other three sICs and higher than that of cervical ultrasound. It is important to note that, the sensitivity and specificity of ultrasound diagnosis for CLNM showed in our manuscript primarily apply to the lateral compartment. Due to the anatomical complexity of the thoracic inlet, ultrasound had limited accuracy in the diagnosis of central CLNM. In a meta‐analysis comprising 4014 patients, ultrasound showed a sensitivity of 0.33 and specificity of 0.93 for central CLNM, and sensitivity of 0.70 and specificity of 0.84 for lateral CLNM.^6^ Among the 71 patients involved in our study, only 2 cases were reported to have suspicious central CLNM on preoperative neck ultrasound, whereas prophylactic central dissection revealed metastasis in nearly half of the patients (30 out of 71). Furthermore, the controversy regarding the performance of prophylactic central neck dissection persists. While the significance for prophylactic central neck dissection for clinically node‐negative (cN0) PTC and for most FTC remains unclear according to the recommendations of ATA guidelines (2015),^17^ it had been recommended in the latest guidelines from the Chinese Society of Endocrinology (2023),^7^ as well as in guidelines of other countries.^8^, ^9^ Therefore, our findings can provide some reference for surgical decision‐making in DTC patients. The higher specificity (91.89%) implies that prophylactic central neck dissection may be more beneficial for cN0 DTC patients with abnormally elevated serum sTIM‐3 levels.

During the exploration of sIC resources, sIC levels in LNs were found to be significantly higher than those in PBMCs, and the level of sTIM‐3 in TILNs from the same patient was higher than that in normal LNs. To our knowledge, this is the first study to report the detection of sIC levels in locoregional lymph nodes of DTC. Moreover, in TILNs with high levels of sTIM‐3, CD4^+^ and CD8^+^ subsets had significantly higher levels of TIM‐3 on the surface than those of nLNs of the same patient. Increased TIM‐3 expression usually reflects the later exhaustion stage of T cells.^11^ Our study and an open‐access database revealed that the transcription factors, Tbx21 and Tcf7, which are involved in the early and intermediate exhaustion stages, were significantly lower in TILNs than in nLNs of the same patient.^18^ However, a difference was found between our results and the open‐access database for Tox, a transcription factor involved in the late exhaustion stage. Based on our results, the transcription level of Tox in TILNs was higher than that in nLNs; however, the open‐access database revealed no difference between them. This difference may be due to the limited number of samples and different detection methods. Tox is uniquely expressed in tumors. Single‐cell transcriptome analysis revealed that Tox is the only exhaustion‐related transcription factor that is ubiquitously expressed in different tumors, such as melanoma and non‐small‐cell lung cancer,^19^ and its expression plays a critical role in the presence of tumor‐specific T cells in the tumor microenvironment.^20^ Therefore, our results consistently suggest that the increased proportion of TIM‐3^+^T lymphocytes in the TILNs of DTC patients is related to their exhaustion status; however, functional experiments are needed. Importantly, the sTIM‐3 secretion levels of T lymphocytes isolated from TILNs were found to increase. Unlike other sICs, such as sGalectin‐9^21^ and sPD‐L1,^22^ the secretion of sTIM‐3 is currently known to be completely dependent on the proteolytic cleavage of ADAM protease, but independent of alternative splicing of mRNA.^23^ Thus, the expression of TIM‐3 on the cell membrane is closely related to the secretion of its soluble form. Therefore, we speculate that TIM‐3^+^ T lymphocytes in TILNs are an important source of sTIM‐3.

Notably, a significant decrease in sTIM‐3 levels was found in the serum of DTC patients after surgical treatment with prophylactic central CLN dissection; this decrease mainly occurred in patients with CLNM. Although previous studies have shown that sICs, such as sPD‐1, remain stable or increase after certain treatments, such as radiotherapy, anti‐EGFR therapy, or immunotherapy, and is associated with better prognosis in tumor patients,^24^, ^25^, ^26^ these treatments are not completely curative and the tumor microenvironment still persists during treatment. As a result, the increase in sIC may be due to the reactivation of tumor‐specific T cells.^27^, ^28^ Compared to other sICs, changes in sTIM‐3 after tumor treatment have rarely been studied. To the best of our knowledge, this is the first report of changes in sTIM‐3 after surgery in patients with DTC. Whether the postoperative decrease in sTIM‐3 implies that it could be applied as a marker of DTC recurrence or metastasis is being investigated in a longer follow‐up study.

In conclusion, our results suggest that sICs, especially sTIM‐3, are effective preoperative predictors of CLNM in DTC patients. The increased levels of sTIM‐3 in the serum of DTC patients with CLNM may be related to TIM‐3 overexpression on the surface of T cells in TILNs. Our study had several limitations. First, due to inaccessibility to tumor samples, we failed to isolate enough lymphocytes from DTC tissue. As a result, we could not analyze the relationship between sIC and tumor‐specific T lymphocytes in the DTC tumor microenvironment. Second, the function of sTIM‐3 is still in the preliminary exploration stage and is controversial.^23^, ^29^ Currently, the effect of sTIM‐3 on anti‐tumor immune activity or its role in DTC progression has not been verified, but will be explored in future studies. Nevertheless, our results suggest the clinical value of sIC, particularly sTIM‐3, in the pre‐operative prediction of DTC. sIC combined with conventional examination methods, such as cervical ultrasound, may further improve the sensitivity and accuracy of CLNM preoperative prediction.

## AUTHOR CONTRIBUTIONS

**Yi Shao:** Conceptualization (lead); investigation (equal); methodology (equal); writing – original draft (equal). **Xinru Gui:** Formal analysis (equal); investigation (lead); methodology (lead). **Yuxin Wang:** Formal analysis (equal); investigation (equal); methodology (equal). **Lei Sheng:** Investigation (equal). **Dong Sun:** Funding acquisition (equal); validation (lead). **Qingdong Zeng:** Investigation (equal). **Huayang Wang:** Conceptualization (lead); funding acquisition (equal); methodology (equal); writing – review and editing (lead).

## FUNDING INFORMATION

This work was supported by grants from Natural Science Foundation of Shandong Province (grant no. ZR2020LZL015 and ZR2022LZL002).

## CONFLICT OF INTEREST STATEMENT

The authors declare no conflict of interest.

## ETHICS STATEMENT

Experiments containing human materials were approved by the Human Research Ethics Committee of the Qilu Hospital of Shandong University (China). All patients have provided consent for sample collection and subsequent analyses.

## Supporting information


Data S1.

## Data Availability

The data used in this analysis are available from the corresponding author upon reasonable request.
