# Supplementary material for: Serum soluble immune checkpoint levels predict cervical lymph node metastasis of differentiated thyroid carcinoma patients
Source: Cancer Med. 2023 Jul 27;12(17):17648–59. doi: 10.1002/cam4.6382 (PMC10524022; doi:10.1002/cam4.6382)
Supplement: Supplementary file 1 — Data S1. [file CAM4-12-17648-s001.docx]

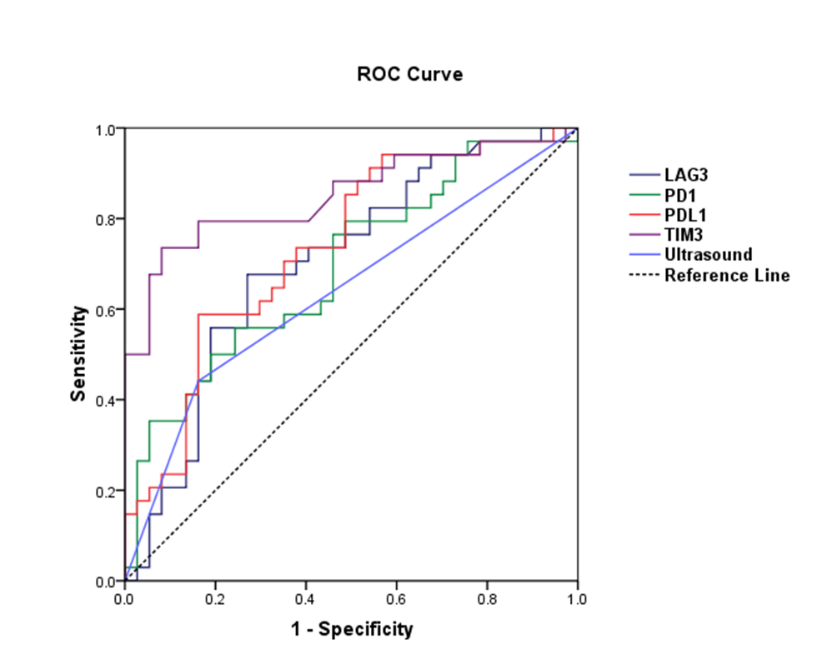


**Supplemental Figure 1. ROC curve of serum sIC values and cervical ultrasound in discriminating CLNM of DTC patients.**

**Supplemental Table 1. Clinical features of DTC patients**

| **Clinical Parameters** | **Value** |
| --- | --- |
| **Gender** |  |
| Male | 19 |
| Female | 52 |
| **Age (year)** | 42.68 (39.96-45.39)^a^ |
| **Ultrasound features** |  |
| TI-RADS score |  |
| IV | 42 |
| V and above | 29 |
| Suspicious CLNM |  |
| No | 50 |
| Yes | 21 |
| **Operation style** |  |
| Thyroidectomy |  |
| Total/Near-total | 27 |
| Lobo‑isthmectomy | 44 |
| LN dissection |  |
| Central only | 54 |
| Central and Lateral | 17 |
| **Thyroid cancer subtype** |  |
| Classical papillary | 66 |
| Follicular variant of papillary | 5 |
| **Maximum tumor diameter (mm)** | 8.44 (7.23-9.65)^a^ |
| **Multifocality** |  |
| Yes | 57 |
| No | 13 |
| Censored | 1 |
| **LN involvement** |  |
| Non | 37 |
| Central only | 20 |
| Lateral | 14 |
| **Average involved LN number^b^** | 5.21 (2.78-7.63)^a^ |
| **TNM stage** |  |
| I | 68 |
| II | 3 |
| **Recurrence risk stratification** |  |
| Low | 38 |
| Intermediate | 29 |
| High | 4 |
| **BRAF^V600E^ status** |  |
| Wild | 7 |
| Mutation | 45 |
| Consored | 19 |
| **Hashimoto's thyroiditis** |  |
| Yes | 28 |
| No | 43 |

a. represented as mean (95%CI)

b. in CLNM (+) patients

**Supplemental Table 2. Multivariate analysis of clinical features and laboratory results in discrimination CLNM of DTC patients**

| **Variants** | **Hazard Ratio (95%CI)** | ***P* value** |
| --- | --- | --- |
| **Age** | 0.08 (0.01-0.64) | **0.03** |
| **Gender** | 2.81 (0.40-21.31) | 0.30 |
| **TI-RADS score** | 1.44 (0.22-8.72) | 0.69 |
| **Suspicious CLNM** | 3.25 (0.52-24.85) | 0.22 |
| **sLAG-3** | 2.41 (0.43-15.68) | 0.32 |
| **sPD-1** | 4.14 (0.65-30.96) | 0.14 |
| **sPD-L1** | 2.47 (0.43-14.76) | 0.30 |
| **sTIM-3** | 48.35 (8.27-499.61) | **<0.01** |
